# Antenatal Perineal Training for Injuries Prevention: Follow Up after Puerperium

**DOI:** 10.3390/medicina60081264

**Published:** 2024-08-05

**Authors:** Federico Villani, Cristian Furau, Barbara Mazzucato, Antonella Cavalieri, Oana Cristina Todut, Victoria Ciobanu, Giuseppe Dodi, Ion Petre

**Affiliations:** 1 Multidisciplinary Doctoral School, “Vasile Goldis” Western University of Arad, 310414 Arad, Romania; vllfrc@gmail.com (F.V.); bisorcaoanacristina@yahoo.com (O.C.T.); 2 Department of Pathophysiology, Faculty of Medicine, “Vasile Goldis” Western University of Arad, 310414 Arad, Romania; vica.ciobanu@gmail.com; 3 The Rehabilitation Therapy of the Pelvic Floor, UniCamillus, Saint Camillus International University of Health and Medical Sciences, 00131 Rome, Italy; babiostri@gmail.com (B.M.); antonella.amaca@gmail.com (A.C.); 4 Doctoral School, “Victor Babes” University of Medicine and Pharmacy, 300041 Timisoara, Romania; petre.ion@umft.ro; 5 Department of Surgical, Oncological and Gastroenterological Sciences, University of Padua, 35128 Padua, Italy; giuseppe.dodi@unipd.it; 6 Department of Functional Sciences, Medical Informatics and Biostatistics Discipline, “Victor Babes” University of Medicine and Pharmacy, 300041 Timisoara, Romania

**Keywords:** antenatal perineal training, stretching balloon, perineal massage, perineal injuries, episiotomy, pelvic floor dysfunctions

## Abstract

*Background and Objectives:* This retrospective analysis investigated the impact of preparation of the pelvic floor for childbirth with stretching balloons and perineal massage on the risk of pelvic floor injuries. *Materials and Methods*: We analyzed 150 primiparous women who accessed private clinics in Padua (Italy) in the period 2019–2023 regarding the rate of perineal trauma and postpartum dysfunction across three groups: the balloon stretching group (BSG, N = 50, 33.3%), the perineal massage group (PMG, N = 39, 26.0%), and the control group (CG, 61, 40.7%). *Results:* Prenatal perineal training had a significant impact on reducing the rate of perineal injury and episiotomy (27.5% in BSG vs. 48.7% in PMG and 68.3% in CG, *p* = 0.008, respectively, 9.8% vs. 26% and 40%, *p* = 0.046) and the duration of the second stage of labor (BSG and PMG had a shorter duration compared to CG with a mean difference of −0.97892 h, *p* < 0.001, respectively, −0.63372 h, *p* = 0.002). Patients who carry out the preparation with the stretching balloon are less likely to develop urinary and anal incontinence and pain during intercourse. Specifically, the rate of urinary incontinence in BSG stands at around 23.5% compared to 43.6% in PMG (*p* = 0.345) and 55% in CG (*p* = 0.034). Dyspareunia in BSG was detected in 11.8% of cases compared to 35.5% in PMG (*p* = 0.035) and 61.7% in CG (*p* < 0.01). Symptomatology inherent to the posterior compartment was reported in 9.8% of cases in BSG vs. 23.11% in PMG (*p* = 0.085) and 33.3% in CG (*p* = 0.03%). *Conclusions*: Stretching balloons and perineal massage can be chosen as tools to prevent and reduce the rates of obstetric trauma during childbirth and to reduce the use of episiotomies as well as protect against the development of dysfunctions of the pelvic floor.

## 1. Introduction

Pelvic floor disorders have a worldwide prevalence ranging from 1.9% to 46.50% [1,2]. It is now known that pregnancy and childbirth represent the main risk factors for the development of pelvic floor pathologies. The stresses that the pelvic floor is forced to bear during pregnancy are associated with the action of a hormone, relaxin, which has the task of reducing abdominal muscle tone to allow distension of the uterus. The loss of tone of the muscles that make up the pelvic floor leads to greater stress on the ligaments and tendons that compose it with an irreversible stretching of these structures. Furthermore, the growth of the fetus during pregnancy involves a postural reorganization of the woman both due to the direct effect of the pelvic organs and abdominal muscles, in relation to the pelvis and the lumbar spine, and due to the volumetric increase of the uterus, bringing the body to a new balance with an increase in lumbar lordosis and the shift of pressures from the pregnant uterus toward the urogenital hiatus. Vaginal birth causes damage to the pelvic floor, which is due in part to stretching of the levator ani muscle to 2.5 times its original length [3]. Childbirth can lead to fascial, muscular, vascular/ischemic and/or neurogenic damage to the pelvic floor [4]. Studies suggest that pudendal nerve compression injuries during childbirth may persist or worsen over time [5].

More than 85% of women who have a vaginal birth suffer some form of perineal trauma, and 60% to 70% receive stitches. In the UK, approximately 23% suffer from dyspareunia at 3 months, 3 to 10% report symptoms of anal incontinence and up to 24% experience urinary incontinence [6,7]. The clinical incidence of third- and fourth-degree lacerations varies considerably, the prevalence is 0.5–3% in Europe and 6–9% in the USA [8]. Occult sphincter lesions affect 35% of primiparous mothers and 44% of multiparous mothers; failure to identify these lesions can lead to the development of symptomatic pictures over time in association with further concomitant risk factors (age, menopause, other births, surgery). The Royal College guidelines on the management of third- and fourth-degree injuries recommend that women perform perineal massage from the 35th gestational week, supported by a professional, as a prevention of perineal trauma and to reduce the risk of obstetric anal sphincter injuries [9]. Nine out of ten women are affected by perineal trauma, and these data suggest that adequate antepartum muscle preparation is not performed. We decided to conduct a retrospective analysis to understand whether the use of techniques, such as perineal massage and balloon stretching, to increase the extensibility of the tissues, leads to a reduction in the prevalence of perineal injuries and associated postpartum morbidities. Therefore, the purpose of this study was to determine whether perineal training performed in the final weeks of pregnancy could lower the frequency and severity of perineal injuries sustained during childbirth as well as to assess the long-term effects of these exercises by monitoring the participants following the puerperium period. The findings of this study may offer insightful information on the advantages of perineal training during pregnancy and its possible contribution to better maternal health outcomes.

## 2. Materials and Methods

### 2.1. Study Protocol

This study is a three-year retrospective analysis evaluating the rate of perineal trauma and pelvic floor dysfunction based on the type of perineal preparation performed during pregnancy. We analyzed the data of 150 women in their first pregnancy who accessed private clinics based in Padua (Italy) from September 2019 to November 2023, for prenatal evaluation, postpartum visit at 40 days and for pelvic floor evaluation within 6 months of delivery. The clinic is a facility specific to pelvic floor pathologies, and pregnant women are referred by other specialists or present spontaneously for an evaluation of the pelvic floor during pregnancy. As a routine, the methods of perineal preparation for birth are presented and explained to women, such as perineal massage or perineal balloon, leaving the woman free to decide whether or not to use one of the techniques respecting a physiological pregnancy. At the first access, the clinic, in addition to the privacy documentation, presents the informed consent to the use of data for scientific research. 

Based on the medical records, the patients were selected who had complete clinical and anamnestic data, which would allow the establishment of the type of perineal preparation during birth, the type of birth and the possible obstetric traumas/episiotomy associated with birth, the evolution of the puerperium, and the presence of symptoms associated with perineal dysfunction (urinary and anal incontinence and pelvic pain) within 6 months of delivery (N = 278). Women with their first child, who had a vaginal delivery and who signed an informed consent for the study and for the processing of personal data were included. The following criteria had to be met to be included in the study: women with singleton pregnancy, over 18 years of age, no previous caesarean section, no pregnancy complications such as preeclampsia, placenta previa, vaginal infections and the threat of premature birth. Patients in the 2 study groups, balloon stretching and perineal massage, respectively, were required to report at postpartum reassessment that they followed the perineal preparation as instructed.

Exclusion criteria were multiparity, fetal macrosomia (neonatal weight ≥ 4500 g), twin pregnancy, uterine malformations, caesarean section, previous surgery or trauma to the perineal area, conditions such as preeclampsia, gestational diabetes or placenta previa, participants at risk or who presented the threat to give birth prematurely, anterior perineal preparation during pregnancy, women with functional pathologies of the pelvic floor prior to pregnancy, women with osteo-articular pathologies in the lumbosacral area and pelvis, active genital infections or sexually transmitted infections, allergies to the materials used in the lengthening balloon or massage lubricants, conditions such as severe varicose veins in the perineal area, coagulation disorders or other medical contraindications to perineal manipulation, lack of informed consent, and for the 2 study groups, those who declared that they did not perform the preparation correctly or discontinuously. 

**Figure 1:**
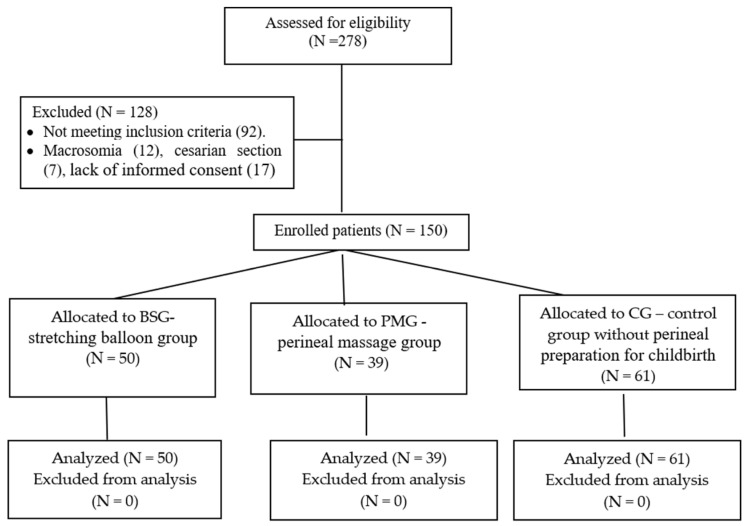
Flow chart of the study.

The methods for perineal preparation included perineal massage and stretching balloon training. Patients who used one of these techniques formed the study groups: balloon stretching group (BSG, N = 50, 33.3%) and perineal massage group (PMG, N = 39, 26.0%). Women who did not use any method of perineal preparation for childbirth made up the control group (CG, 61, 40.7%) (Figure 1).

Using the OpenEpi program and setting the test’s power at 80% [10], the sample size could be determined given the total number of patients (N = 278) taken into consideration for our study. This led to the representative sample size of 104 subjects.

From the medical records of the patients included in the study, data related to birth were centralized (type of pregnancy, type of preparation performed antepartum, mode of delivery—spontaneous, induced, operative, use of epidural analgesia, use of Kristeller maneuver, type of perineal trauma—laceration, episiotomy, expulsion period times, maternal position during birth, neonatal weight), and puerperal course (regular or complicated) as well as data related to pelvic floor dysfunctions in the first 6 months after birth (urinary incontinence—stress, urgency, mixes, fecal incontinence, and pain during intercourse. All births were assisted in specialized clinics/hospitals by qualified medical personnel (midwife/doctor) in accordance with internal protocols and national specialty guidelines.

### 2.2. Birth Preparation Methods

The birth preparation techniques were recommended, explained in detail to the patients, and supervised by specialists from the clinics. 

Stretching balloon training consisted of using a device equipped with an anatomic silicone balloon, a manual pump, a manometer, a release valve, and a flexible connecting tube; this device allows the pelvic floor muscles to be stretched in preparation for birth to gradually train their extensibility. The lubricated balloon is inserted halfway into the vagina and inflated slowly until a slight sensation of tension is felt, which indicates relaxation of the tissues. The inflated balloon is left in the vagina for about 10 min. At the end of the exercise, the pelvic floor muscles relax, thus encouraging a gradual expulsion of the balloon from the vagina. As training progresses, the diameter of the balloon should increase in subsequent sessions. The technique was performed daily, alternating 2 sessions per day of approximately 10 min with one session per day for approximately 15–20 min, starting from the 35th week of gestation until term.

Perineal massage was performed by the patients and consisted of inserting the lubricated thumb into the vagina up to the first phalanx and sliding it clockwise and counterclockwise, applying constant downward and lateral pressure for several minutes until a tingling sensation or numbness. After that, an outward pressure was applied for about twenty seconds, then returning to the basal tone (center, right, and left). The technique was applied daily, for about 10 min a day. starting from the 35th week of gestation until the end of pregnancy.

### 2.3. Statistical Analysis

The database was collected in Microsoft Excel and statistical analysis was performed using JASPv18.1. Frequency tables and descriptive statistical analysis were used to describe the database. For numerical variables, the Shapiro–Wilk test was used to determine the data distribution. The chi-square test was employed to compare proportions, and non-parametric tests (Mann–Whitney for two groups, and Kruskal–Wallis for more than two groups) were utilized to compare variables that did not have a normal distribution. A correlation model was used to examine the link between the data, and the Spearman parameter was computed to test the dependence of the data. We used α = 0.05 as the level of significance for the whole investigation.

## 3. Results

Among the selected patients, 150 fulfilled the requirements to be included in this retrospective analysis. Most patients (over 70%) were in the 30–34 and 35–39 age groups and resided in urban areas (90%). There were 74 cases (49.3%) of spontaneous births at term, 34 cases (22.7%) of induced births at term, and 25 (16.7%) cases of ventouse deliveries. Additionally, spontaneous births were observed at 36–39 weeks of gestation (12.3%). Episiotomy was used in 39 cases (26%). In the case of patients with perineal tears, grade 2 lacerations were the most frequent (24%), which were followed by grade 1 lacerations (16%). The postpartum period, or puerperium, was reported as regular in 130 cases (86.7%). However, there were 3 cases (2%) of peri sutural hematoma, 3 cases (2%) of postpartum hemorrhage, 10 cases (6.7%) of perineal pain and tension, 2 cases of congested hemorrhoid and suture pain, and 2 cases of suture diastasis. The most common pelvic floor dysfunctions were urinary incontinence of various types (42.7%), which was followed by dyspareunia (38%) and anal incontinence (22.7%). Table 1 lists the pertinent patient characteristics that were examined for this research.

The analysis of differences in the frequency of perineal injuries at birth and pelvic dysfunction within 6 months, by age categories, indicated that there are no significant differences between the age groups and the rate of the tested parameters, except for the presence of dyspareunia, which was significantly more frequent in the age groups 40–44 (76.5%), followed by 45–49 (66.7%) and from the 25–29 group (52.6%). The lowest frequency of dyspareunia was recorded in the 35–39 age group (22.0%) (Table 2).

Data analysis regarding the age of patients who received epidural anesthesia or suffered perineal trauma and pelvic floor dysfunction showed that these were, in general, older than those without these conditions without significant differences (*p* > 0.05) (Table 3).

**Figure 2:**
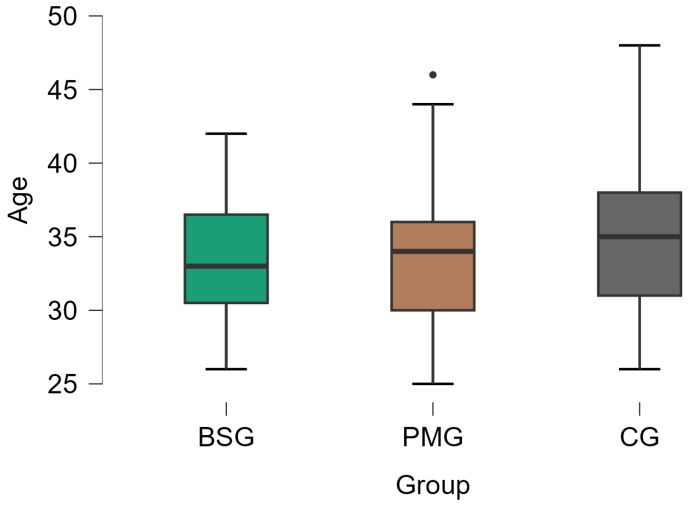
The age distribution by groups. BSG—balloon stretching group, PMG—perineal massage group, CG—control group.

In order to assess the impact of perineal preparation for injury prevention, a detailed statistical analysis was conducted between groups. The mean age of the participants was 34.19 years with a standard deviation of 4.51 years and with an interval between 25 and 48 years. The average age was relatively homogeneous between the 3 groups (Figure 2) without significant differences (33.51 years, SD = 3.89 for BSG; 33.97 years, SD = 4.65 for PMG; 34.9 years, SD = 4.86 for CG, statistic =2.739, *p* = 0.254).

Analysis of the data related to the parameters tested at birth indicates significant differences (*p* < 0.05) between the groups at the level of the type of birth, maternal position, the application of the Kristeller maneuver as well as for episiotomy and perineal tears (Table 4). 

At the level of the intervention groups (BSG vs. PMG), the lack of perineal injury and episiotomy was significantly more frequent in the BSG group (72.5% vs. 51.3%, *p* = 0.008, respectively, 90.2% vs. 74%, *p* = 0.046), indicating a positive impact of the balloon stretching intervention compared to perineal massage on these parameters. BSG vs. CG analysis indicated significant differences (*p* < 0.05) in all tested parameters with a higher frequency of positive results in BSG. In the PMG group, the free position of the fetus was significantly more frequent than in the control group (25.6% vs. 0%, *p* < 0.001), while the application of the Kristeller maneuver was significantly less (10.3% vs. 28.2%, *p* = 0.032) as shown in Table 5.

**Figure 3:**
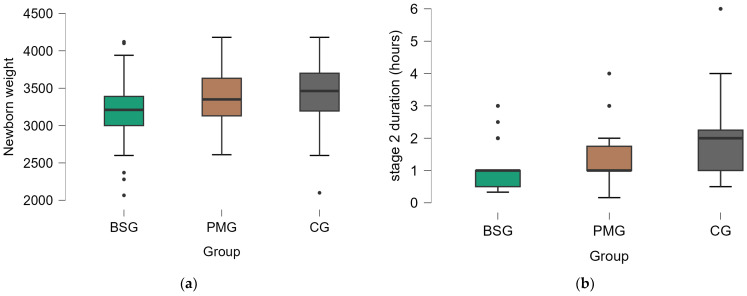
The distribution of variables by groups presented using boxplots: (**a**) newborn weight, (**b**) second stage of labor duration. BSG—balloon stretching group, PMG—perineal massage group, CG—control group.

The mean newborn weight was 3205.18 g (SD = 434.46) in BSG, 3366.18 g (SD = 370.43) in PMG, and 3430.43 g (SD = 415.06) in CG (Figure 3a). BSG had a lower mean newborn weight compared to CG with a mean difference of −225.25686 g, which was statistically significant (*p* = 0.014). PMG had a lower mean newborn weight compared to CG, but the difference of −64.25385 g was not statistically significant (*p* = 1.000). CG had a significantly higher mean newborn weight compared to Group 1 with a mean difference of 225.25686 g (*p* = 0.014).

The mean duration of the second stage of labor was 1.01 h (SD = 0.67) in Group 1, 1.36 h (SD = 0.74) in PMG, and 1.99 h (SD = 1.12) in Group 3. BSG had a significantly shorter duration of the second stage of labor compared to CG with a mean difference of −0.97892 h (*p* < 0.001). PMG also had a significantly shorter duration of the second stage of labor compared to CG with a mean difference of −0.63372 h (*p* = 0.002). CG had a significantly longer duration of the second stage of labor compared to BSG with a mean difference of 0.97892 h (*p* < 0.001). CG had a significantly longer duration of the second stage of labor compared to PMG with a mean difference of 0.63372 h (*p* = 0.002).

**Figure 4:**
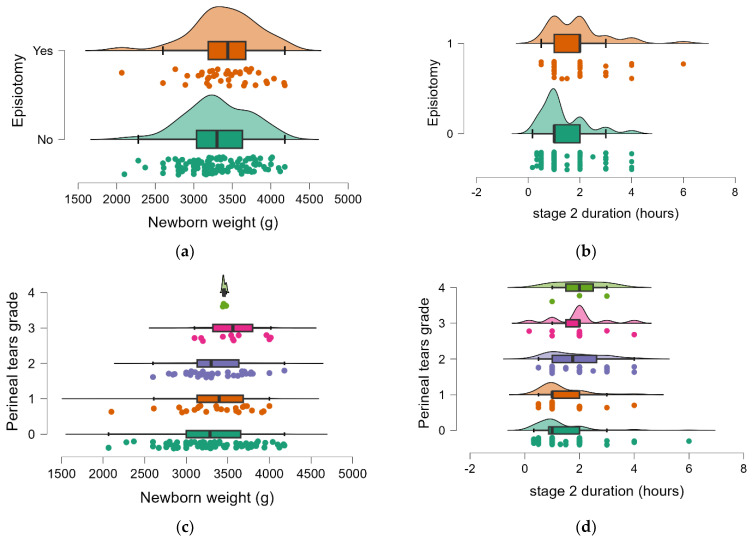
The distribution of newborn weight and second stage of labor presented using raincloud plots: EG (**a**,**b**); PTG (**c**,**d**).

To determine the possible impact of weight of the newborn and the second stage of labor duration on perineal trauma (episiotomy and perineal tears), two distinct groups were formed based on the use of episiotomy (EG—yes/no) and depending on perineal tears (PTG—laceration grade 0 to 4). The findings of the analysis showed that while the length of the second stage of labor is significantly associated with the tested parameters, the newborn’s weight did not significantly affect the perineal damage at birth (*p* > 0.05). The results of the analysis are presented in Table 6 and Figure 4a–d.

**Figure 5:**
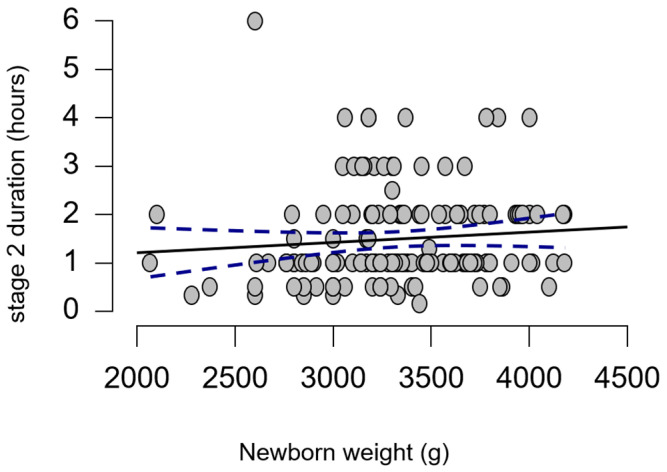
Correlation plot between the weight of the newborn and the second stage of labor.

Using a correlation model, we examined any potential relationships between the weight of the newborn and the second stage of labor. The results showed no significant correlations (Spearman’s rho = 0.157, *p* = 0.055), as revealed in Figure 5. 

At the level of puerperium, the data analysis indicates a higher frequency of regular puerperium in BSG (96.1%) compared to PMG (87.2%) and CG (86.7%) but without significant differences (*p* > 0.05) (Table 7). In BSG, only perisutural hematoma and postpartum hemorrhage appeared as complications with a frequency of 2% each compared to PMG (2.6%) and CG (1.7%). At the BSG and CG level, other complications also appeared: perineal pain and tension (5.1% in PMG, 13.3% in CG), congested hemorrhoid and suture pain (2.6% in PMG, 1.7% in CG), and diastasis sutures, which were present only in CG in 3.3% of cases.

At six months after birth, the frequency of pelvic floor dysfunctions was significantly different between groups (*p* < 0.05) regardless of their type (Table 8). 

The comparison of the two treatment groups (BSG vs. PMG) determined significant differences only in the case of dyspareunia, which was significantly more frequent in the PMG group (χ^2^ = 8.586, *p* = 0.035). Urinary incontinence of different types was more frequent in CG with significant differences compared to BSG (χ^2^ = 8.625, *p* = 0.034) and insignificant differences compared to PMG (χ^2^ = 2.827, *p* = 0.419). In the case of fecal incontinence, the data also indicate a higher frequency in the control group with significant differences compared to BSG (χ^2^ = 8.746, *p* = 0.003 *) and insignificant differences compared to PMG (χ^2^ = 1.200, *p* = 0.273). Dyspareunia was significantly more frequent in the control group both compared to BSG (χ^2^ = 30.335, *p* < 0.001 *) and PMG (χ^2^ = 7.057, *p* = 0.029 *) (Table 9).

A multivariate logistic regression analysis was used to assess the influence of the parameters tested at birth and of perineal preparation on the probability of dyspareunia in participants (see Table 10). According to the results, there is a significant correlation (χ^2^ (122) = 48.970, *p* < 0.001) between the predictor variables and the dyspareunia. The most important predictors were the use of episiotomy, pelvic damage and the lack of perineal preparation. 

**Figure 6:**
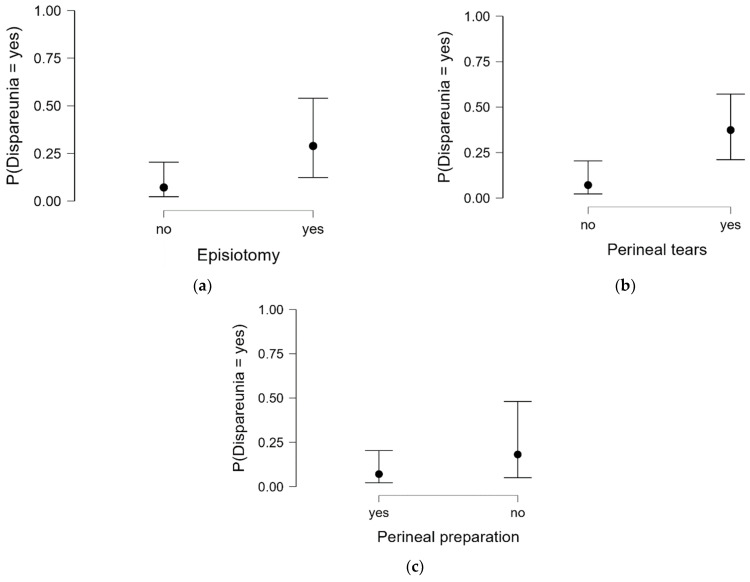
The logistic regression model for significant predictors of dyspareunia: (**a**) episiotomy, (**b**) perineal tears, and (**c**) perineal preparation.

The probability that dyspareunia will occur increased with the increase in the rate of episiotomy, the presence of perineal tears and the lack of perineal preparation (Figure 6).

## 4. Discussion

Pregnancy and childbirth are among the main risk factors for developing pelvic floor dysfunction. In addition to hormonal, cardiovascular, and psychological factors of pregnancy, the perineum can suffer various types of damage with the appearance of symptoms such as urinary and fecal incontinence, dyspareunia and chronic pelvic pain [11,12]. Also, maternal age, parity, maternal position during birth, advanced gestational age, birth weight, fetal malpresentations and malposition, instrumental vaginal delivery, precipitous delivery, and prolongation of the second stage of labor are associated with increased growth risk of perineal trauma [13].

The guidelines for clinical practice drawn up by the “Organisme professionnel des médecins exerçant la gynécologie et l’obstétrique en rance” (CNGOF) define how a perineal preparation in pregnancy can prevent perineal lesions and dysfunctional symptoms that occur in the puerperium such as incontinence, dyspareunia and pelvic pain [14,15]. Prenatal perineal massage, the use of the Epi-No device, and exercises to educate the pelvic floor muscles are some of the interventions that have been reported to potentially reduce the incidence of postnatal perineal injury or dysfunction [14,15]. However, one important aspect of PFD prevention is prepartum patient counseling about pelvic floor structure and functioning as well as how to prevent PFD throughout pregnancy and after birth [16,17].

This retrospective study aimed to evaluate the effectiveness of antenatal perineal training with stretching balloons and perineal massage in reducing perineal trauma during vaginal deliveries. Our findings indicate a significant reduction in the incidence of severe perineal tears (third and fourth degree) and the need for episiotomy among women who engaged in antenatal perineal training compared to those who did not. Additionally, participants reported lower levels of postpartum perineal pain and dyspareunia. One hundred and fifty primiparous women with vaginal delivery were analyzed regarding perineal damage at birth, puerperium evolution and incidence of PDF in the first 6 months after delivery, depending on the method of perineal preparation. The average age was 34.19 years, and they were mostly women aged between 30–39 years. The results did not indicate an association between age and the parameters tested in this study apart from dyspareunia. Dyspareunia, the experience of pain during sexual intercourse, is a prevalent concern among postpartum individuals [18]; 38% of patients reported the presence of dyspareunia after childbirth in aur study. The analysis revealed significant differences in dyspareunia across different age categories, with more frequent incidence in extreme age categories (40–49 years and 25–29 years). This suggests that age-related factors may play a role in influencing postpartum sexual function, which is confirmed by other studies [18]. Moreover, in this study, based on a multivariate regression analysis, the most important predictors for dyspareunia were the lack of perineal preparations, the application of epidural anesthesia, the use of an episiotomy, and perineal damage.

Each group was assessed based on various parameters related to birth type, perineal tears, episiotomy, Kristeller maneuver, maternal position at birth, epidural anesthesia, puerperium, the duration of the second stage of labor, and newborn weight. By analyzing and comparing the two study groups, BSG and PMG and the control group, we can state that those who chose to carry out a perineal preparation had a lower probability of experiencing perineal outcomes compared to the group where no treatment was carried out.

The best results were obtained in the BSG group, where perineal injury and episiotomy had a significantly lower frequency compared to PMG and CG (27.5% vs. 48.7% and 68.3%, *p* = 0.008, respectively 9.8% vs. 26%, and 40% *p* = 0.046), indicating a positive impact of the balloon stretching intervention compared to perineal massage and no pelvic preparation on these parameters. Perineal massage also reduced the rate of perineal injuries and episiotomy, but the differences were insignificant compared to the control group (*p* > 0.05). These results are similar to those obtained in other studies that indicate that preventive perineal preparation seems to reduce the rate of episiotomy [19] and increase the rate of intact perineum [20].

The most difficult part of childbirth, for both women and midwives, is the second stage of labor. Cutting the length of the second stage of labor short is crucial because the literature currently in publication indicates that prolonging the period may raise the risk of difficulties for both the mother and the newborn [21]. The analysis of data from this study indicated that prenatal perineal training had a significant impact on reducing the duration of the second stage of labor compared to the control group. BSG had a shorter duration with a mean difference of −0.97892 h (*p* < 0.001) and PMG had a shorter duration of the second stage of labor compared to CG with a mean difference of −0.63372 h (*p* = 0.002). This fact determined a reduction in the rate of episiotomy and perineal tears in these groups. Previous studies suggest that perineal massage can be used to reduce perineal injuries in primiparous women both before and during the second stage of labor [22,23,24]. Antenatal perineal massage can significantly reduce the rates of episiotomies and severe perineal tears (third- and fourth-degree). A systematic review by Aasheim et al. (2017) [25] found that perineal massage starting from 35 weeks of gestation reduced the likelihood of perineal tears in nulliparous women by about 10%. This reduction was more pronounced in women who had not given birth before.

Our study adds to this body of evidence by confirming these benefits in a real-world, retrospective cohort. Choosing outcome measures that are universally relevant (e.g., rates of perineal tears, postpartum recovery) ensures that the findings are meaningful across different populations and settings.

In the literature, there is still very little data relating to birth training, and the studies published so far have not found a significant benefit [26], even if the method with which they were carried out is questionable and there is no high statistical power. In fact, the centers taking part in the study have a high rate of episiotomy, and it seems that the instructions in the user manual for using the device were not respected as well as not having acquired data on postpartum dysfunctions. Another study that evaluated prenatal pelvic floor training with a vaginal balloon device found no association between improved pelvic health outcomes and the use of the antenatal training device in nulliparous women who had a vaginal birth at term. Still, it might lessen the number of episiotomies performed [27].

In one more recent study, perineal massage and balloon stretching were found to improve perineal muscle extensibility when given in multiple sessions to primiparous women starting at the 34th week of gestation, which is very helpful in preventing child trauma during labor [28].

In the study we conducted, however, balloon and perineal massage prevented perineal trauma and postpartum dysfunction with greater benefit for women belonging to BSG. The two groups in question, however, are not comparable from the point of view of objectivity, since the perineal massage was carried out by individual patients without the supervision of a specialized operator; consequently, the correct execution of the massage at home is not ensured. In contrast, the stretching balloon is a medical device used following the relevant user manual with specific instructions; consequently, the data relating to BSG are more objective.

As regards the data relating to pelvic floor disorders reported by women in the various study groups, it emerged that those who carried out the preparation with the stretching balloon (BSG) were less likely to develop urinary and anal incontinence and pain during intercourse. Specifically, the rate of urinary incontinence in BSG stands at around 23.5% compared to 43.6% in the PMG (*p* = 0.345) and 55% in CG (*p* = 0.034). Dyspareunia in BSG was detected in 11.8% of cases compared to 35.5% in PMG (*p* = 0.035) and 61.7% of CG (*p* < 0.01). Symptomatology inherent to the posterior compartment was reported in 9.8% of cases in BSG vs. 23.11% in PMG (*p* = 0.085) and 33.3% in CG (*p* = 0.03%). Long-term follow-up studies suggest that antenatal perineal training can mitigate some postpartum morbidities, such as urinary incontinence and pelvic floor dysfunction. Women who engaged in perineal massage reported fewer instances of pelvic floor weakness and related complications in the months following childbirth [23,29].

The beneficial effects of perineal training can be attributed to several physiological mechanisms. Stretching balloons and perineal massage likely increase the elasticity of perineal tissues, enhancing their ability to stretch during childbirth and thereby reducing the likelihood of tears. This hypothesis is supported by biomechanical studies that demonstrate improved perineal tissue compliance following regular stretching and massage [29]. Moreover, the increased awareness and control over pelvic floor muscles developed through these exercises may contribute to better management of the birthing process.

A systematic review of 3125 studies found that many women have a gap in knowledge about pelvic floor muscle dysfunction that leads to a lack of understanding of treatment options and risk factors for these disorders [30]. The first form of prevention is to provide information to pregnant women and make them aware of the effect that pregnancy and childbirth have on the pelvic floor and educate them on correct behavioral lifestyles, hence the importance of obstetric counseling and accompanying courses at birth. Pregnancy and childbirth are physiological phenomena that can lead to damage to the pelvic floor structures. It is of fundamental importance for the overall health of the pelvic floor to advise the woman to carry out an assessment of the pelvic floor to check her state of health for personalization of the intervention in preparation for the birth event and to identify pregnant women with risk factors through use of the perineal card. Perineal damage cannot always be avoided but can be limited by adopting preventive strategies and integrated therapies.

The retrospective nature of the study provides insights into the practical application and effectiveness of perineal training in routine antenatal care and the detailed categorization and analysis of the groups provides insight into the distribution of perineal trauma and postpartum dysfunction across the three groups. These findings underscore the importance of individualized care approaches that account for age-related factors, obstetric characteristics, and postpartum concerns to optimize the well-being of postpartum individuals and their newborns. This practical approach provides actionable insights for healthcare providers and policymakers. Choosing outcome measures that are universally relevant (e.g., rates of perineal tears, postpartum recovery) ensures that the findings are meaningful across different populations and settings. However, the study has some limitations, the most important of which are determined by the retrospective design and the small number of patients included. As a retrospective study, there is an inherent risk of selection bias and confounding variables that could influence the outcomes. Although we adjusted for known confounders, unmeasured factors may still affect the results. Another limitation of our study is that the data regarding the antepartum pelvic floor health status of the women in the sample are not known, and validated questionnaires were not given to the women to assess whether the dysfunctions were already present during pregnancy or even before pregnancy. Even with the constraints that come with retrospective studies, the results support the body of research and highlight the possible advantages of these practices. Therefore, healthcare providers should consider recommending antenatal perineal training with stretching balloons or perineal massage to pregnant women, particularly those at higher risk of severe perineal trauma. Educating pregnant women about the benefits and techniques of perineal training can enhance adherence and effectiveness. Providing instructional materials and support during antenatal visits can facilitate this process.

Future studies with a follow-up, long-term cohort design, including more diverse populations, are needed to support these results. Conducting well-designed RCTs can help establish causality and further validate the benefits of antenatal perineal training. Including more diverse populations in future studies can enhance the generalizability of the findings and address potential disparities in maternal health outcomes. By addressing these practical implications and future research directions, the field can advance toward a more effective prevention of perineal injuries and improved maternal health outcomes.

## 5. Conclusions

Considering the data collected, stretching balloons and perineal massage can be chosen as tools to prevent and reduce the rates of obstetric trauma during childbirth, reduce the use of episiotomy, and protect from the development of dysfunctions of the pelvic floor. Through our comprehensive approach, which compares the perineal preparation techniques both with each other and with standard care, and evaluates immediate results (rate and severity of perineal injuries during childbirth) and long-term results (pelvic floor function), this study provides a comprehensive understanding of the effectiveness of these interventions and a more complete picture of their benefits. We believe it is interesting that further large-scale studies using the correct lines of use of these techniques can be developed to accredit the concept of prevention as a useful and functional practice for the quality of life of women after childbirth.

## Figures and Tables

**Table 1:** Patients’ characteristics.

Parameters		Total (N = 150)	
		N	%
Age_category	25–29 years	19	12.7
	30–34 years	61	40.7
	35–39 years	50	33.3
	40–44 years	17	11.3
	45–49 years	3	2.0
Environment	urban	135	90.0
	rural	15	10.0
Birth type	Spontaneous at term	74	49.3
	Induced at term	34	22.7
	Ventouse delivery	25	16.7
	Spontaneous 39 weeks	3	2.0
	Spontaneous 38 weeks	7	4.7
	Spontaneous 37 weeks	5	3.3
	Spontaneous 36 weeks	2	1.3
Perineal tears	Laceration grade 0	76	50.7
	Laceration grade 1	24	16.0
	Laceration grade 2	36	24.0
	Laceration grade 3	11	7.3
	Laceration grade 4	3	2.0
Episiotomy	No	111	74.0
	Yes	39	26.0
Kristeller maneuver	No	123	82.0
	Yes	27	18.0
Maternal position at birth	Free	35	23.3
	Lithotomy	113	75.3
	Crouched	2	1.3
Epidural anesthesia	No	92	61.3
	Yes	58	38.7
Puerperium	Regular	130	86.7
	Peri sutural hematoma	3	2.0
	Postpartum hemorrhage	3	2.0
	Perineal pain and tension	10	6.7
	Congested hemorrhoid and suture pain	2	1.3
	Suture diastasis	2	1.3
Urinary incontinence	No	86	57.3
	Episodic	12	8,0
	Mixt	6	4,0
	SUI	46	30,7
Anal incontinence	No	116	773
	Yes	34	22.7
Dyspareunia	No	73	48.7
	Unrated	18	12.0
	Present before birth	2	1.3
	Yes	57	38.0

SUI—stress urinary incontinence.

**Table 2:** Frequency of the parameters in case of age category.

		Age Category (Years)										χ^2^	*p* ^1^
		25–29 (N = 19)		30–34 (N = 61)		35–39 (N = 50)		40–44 (N = 17)		45–49 (N = 3)			
		N	%	N	%	N	%	N	%	N	%		
Perineal tears	0	11	57.9	32	52.5	23	46.0	8	47.1	2	66.7	17.311	066 0.386
	1	1	5.3	13	21.3	6	12.0	4	23.5	0	0.0		
	2	5	26.3	14	23.0	13	26.0	4	23.5	0	0.0		
	3	2	10.5	1	1.6	7	14.0	0	0.0	1	33.3		
	4	0	0.0	1	1.6	1	2.0	1	5.9	0	0.0		
Episiotomy	No	13	68.4	52	85.2	35	70.0	10	58.8	1	33.3	9.347	0.053
	Yes	6	31.6	9	14.8	15	30.0	7	41.2	2	66.7		
Epidural anesthesia	No	12	63.2	38	62.3	31	62.0	11	64.7	0	0.0	4.900	0.298
	Yes	7	36.8	23	37.7	19	38.0	6	35.3	3	100.0		
Urinary incontinence	No	8	42.1	40	65.6	29	58.0	7	41.2	2	66.7	12.752	0.387
	Episodic	3	15.8	5	8.2	4	8.0	0	0.0	0	0.0		
	Mixt	1	5.3	2	3.3	3	6.0	0	0.0	0	0.0		
	SUI	7	36.8	14	23.0	14	28.0	10	58.8	1	33.3		
Fecal incontinence	No	16	84.2	50	82.0	34	68.0	14	82.4	2	66.7	4.184	0.382
	Yes	3	15.8	11	18.0	16	32.0	3	17.6	1	33.3		
Dyspareunia	No	7	36.8	33	54.1	28	56.0	4	23.5	1	33.3	24.981	0.015 *
	Unrated	1	5.3	7	11.5	10	20.0	0	0.0	0	0.0		
	BB	1	5.3	0	0.0	1	2.0	0	0.0	0	0.0		
	Yes	10	52.6	21	34.4	11	22.0	13	76.5	2	66.7		

^1^ Chi-square test, * significant values, SUI—stress urinary incontinence.

**Table 3:** The differences between the patients’ age and the presence of tested variables.

Parameters	Mean Age (Years)		*p* ^1^
	Yes	No	
Epidural anesthesia	34.7	33.9	0.429
Perineal tears			
Laceration grade 1	34.7	33.9	0.481
Laceration grade 2	34.1	33.9	0.731
Laceration grade 3	35.8	33.9	0.427
Laceration grade 4	36.0	33.9	0.432
Episiotomy	35.3.	33.8	0.089
Urinary incontinence	33.6	34.0	0.261
Episodic	32.4	34.0	0.219
Mixt	33.3	34.0	0.981
SUI	35.2	34.0	0.189
Fecal incontinence	34.9	34.0	0.230
Dyspareunia	33.5	34.1	0.164
Unrated	34.0	34.1	0.984
Present before birth	32.0	34.1	0.563
Yes	34.4	34.1	0.914

^1^ Mann–Whitney U test, SUI—stress urinary incontinence.

**Table 4:** The frequency tables pointing out the birth results by groups.

Birth Results		BSG (N = 50)		PMG (N = 39)		CG (N = 61)		χ^2^	*p* ^1^
		N	%	N	%	N	%		
Birth type	Spontaneous at term	31	60.8	23	59.0	20	33.3	11.305	0.004 *
	Induced at term	9	17.6	10	25.6	15	25.0		
	Ventouse delivery	3	5.9	6	15.4	16	26.7		
	Spontaneous 39 weeks	1	2.0	0	0.0	2	3.3		
	Spontaneous 38 weeks	4	7.8	0	0.0	3	5.0		
	Spontaneous 37 weeks	2	3.9	0	0.0	3	5.0		
	Spontaneous 36 weeks	1	2.0	0	0.0	1	1.7		
Maternal position	Free	25	49.0	10	25.6	0	0.0	34.317	<0.001 *
	Lithotomy	25	49.0	29	74.4	59	98.3		
	Squatting position	1	2.0	0	0.0	1	1.7		
Kristeller maneuver	No	45	88.2	35	89.7	43	71.7	7.220	0.027 *
	Yes	6	11.8	4	10.3	17	28.3		
Epidural anesthesia	No	32	62.7	29	74.4	31	51.7	5.163	0.076
	Yes	19	37.3	10	25.6	29	48.3		
Perineal tears	No	37	72.5	20	51.3	19	31.7	23.239	<0.001 *
	Laceration grade 1	9	17.6	4	10.3	11	18.3		
	Laceration grade 2	5	9.8	11	28.2	20	33.3		
	Laceration grade 3	0	0.0	4	10.3	7	11.7		
	Laceration grade 4	0	0.0	0	0.0	3	5.0		
Episiotomy	No	46	90.2	29	74.4	36	60.0	12.981	0.002 *
	Yes	5	9.8	10	25.6	24	40.0		

^1^ Chi-square test, * significant values. BSG—balloon stretching group, PMG—perineal massage group, CG—control group.

**Table 5:** Statistics for the frequency differences on the tested parameters between groups.

Variable	BSG vs. PMG		BSG vs. CG		PMG vs. CG	
	χ^2^	*p* ^1^	χ^2^	*p* ^1^	χ^2^	*p* ^1^
Birth type	8.794	0.195	12.798	0.046 *	10.786	0.095
Maternal position	1.365	0.054	38.248	<0.001 *	173.563	<0.001 *
Kristeller maneuver	0.051	0.821	4.607	0.032 *	4.632	0.032 *
Perineal tears	111.834	0.008 *	24.417	<0.001 *	5.517	0.238
Episiotomy	3.991	0.046 *	13.024	<0.001 *	2.161	0.142

^1^ Chi-square test, * significant values, BSG—balloon stretching group, PMG—perineal massage group, CG—control group.

**Table 6:** The differences for newborn weight and second stage of labor duration, depending on perineal trauma.

Groups		N	%	Newborn Weight (g)				Second Stage of Labor (Hours)			
				Mean	SD	Statistics	*p*	Mean	SD	Statistics	*p*
EG	Yes	39	26.0	3404.026	429.681	1863.500 ^1^	0.198 ^1^	1.918	1.147	1429.500 ^1^	<0.001 * ^1^
	No	111	74.0	3313.640	415.693			1.345	0.879		
PTG (Laceration grade)	0	76	50.7	3291.039	453.294	4.560 ^2^	0.335 ^2^	1.302	0.989	15.423 ^2^	0.004 * ^2^
	1	24	16.0	3357.500	449.125			1.354	0.827		
	2	36	24.0	3341.694	352.869			1.819	0.972		
	3	11	7.3	3563.727	327.232			1.924	1.016		
	4	3	2.0	3456.667	20.817			2.000	1.000		

^1^ Mann–Whitney U test, ^2^ Kruskal–Wallis test, * significant values, EG—episiotomy group, PTG—perineal tears group.

**Table 7:** The frequency tables pointing out the puerperium results by groups.

Puerperium	BSG (N = 50)		PMG (N = 39)		CG (N = 61)		χ^2^	*p* ^1^
	N	%	N	%	N	%		
Regular	49	96.1	34	87.2	47	78.3	12.906	0.229
Perisutural hematoma	1	2.0	1	2.6	1	1.7		
Postpartum hemorrhage	1	2.0	1	2.6	1	1.7		
Perineal pain and tension	0	0.0	2	5.1	8	13.3		
Congested hemorrhoid and suture pain	0	0.0	1	2.6	1	1.7		
Suture diastasis	0	0.0	0	0.0	2	3.3		

^1^ Chi-square test,. BSG—balloon stretching group, PMG—perineal massage group, CG—control group.

**Table 8:** The frequency tables pointing out the pelvic floor dysfunctions.

Pelvic Floor Dysfunctions		BSG (N = 50)		PMG (N = 39)		CG (N = 61)		χ^2^	*p* ^1^
		N	%	N	%	N	%		
Urinary incontinence	No	37	72.5	22	56.4	27	45.0	8506	0.014 *
	Episodic	3	5.9	3	7.7	6	10		
	Mixt	1	2.0	3	7.7	2	3.3		
	SUI	10	19.6	11	28.2	25	41.7		
Fecal incontinence	No	46	90.2	30	76.9	40	66.7	8654	0.013 *
	Yes	5	9.8	9	23.1	20	33.3		
Dyspareunia	No	35	68.6	21	53.8	17	28.3	25,375	<0.001 *
	Minim	0	0.0	0	0.0	0	0.0		
	Unrated	8	15.7	4	10.3	6	10.0		
	Present before birth	2	3.9	0	0.0	0	0.0		
	Yes	6	11.8	14	35.9	37	61.7		

^1^ Chi-square test, * significant values. BSG—balloon stretching group, PMG—perineal massage group, CG—control group, SUI—stress urinary incontinence.

**Table 9:** Statistics for the frequency differences on the tested parameters.

Variable	BSG vs. PMG		BSG vs. CG		PMG vs. CG	
	χ^2^	*p* ^1^	χ^2^	*p* ^1^	χ^2^	*p* ^1^
Urinary incontinence	3.320	0.345	8.625	0.034 *	2.827	0.419
Fecal incontinence	2.964	0.085	8.746	0.003 *	1.200	0.273
Dyspareunia	8.586	0.035 *	30.335	<0.001 *	7.057	0.029 *

^1^ Chi-square test, * significant values, BSG—balloon stretching group, PMG—perineal massage group, CG—control group.

**Table 10:** Significant predictors of dyspareunia.

Predictor Factors	Regression Coefficients	OR	*p*
(Intercept)	−0.299	0.741	0.871
Newborn weight	−0.001	0.999	0.125
Stage 2 duration (hours)	0.392	1.480	0.193
Episiotomy (yes)	1.666	5.289	0.014 *
Perineal tears (yes)	2.050	7.768	0.001 *
Epidural anesthesia (yes)	0.622	1.862	0.198
Kristeller maneuver (yes)	0.099	1.104	0.866
Perineal preparation (no)	1.064	2.898	0.026 *

Note. Dyspareunia level ‘yes’ coded as class 1, * significant values.

## Data Availability

The original contributions presented in the study are included in the article; further inquiries can be directed to the corresponding author/s.
